# IgG4-Related Disease of Mastoid Presenting as Headache: A Case Report

**DOI:** 10.7759/cureus.65583

**Published:** 2024-07-28

**Authors:** Sanjay M Khaladkar, Ankita Pandey, Sravya Julakanti, Sayali Paidlewar, Ojasvi Sharma

**Affiliations:** 1 Radiodiagnosis, Dr. D.Y. Patil Medical College, Hospital and Research Centre, Pune, IND

**Keywords:** igg4 -related disease, cerebral venous sinus thrombosis (cvst), pachymeningitis, mastoiditis, igg4 disease

## Abstract

IgG4-related disease (IgG4-RD) is a complex multi-system inflammatory disorder that can affect various organs in the body. This condition is characterized by elevated levels of immunoglobulin G subclass 4 (IgG4) and the presence of specific histopathological features. While neurological involvement is not as common as in other organs, when it occurs, it can lead to hypertrophic pachymeningitis and hypophysitis.

Here, we present a case of a 53-year-old male with right-sided hemicranial headache and diplopia. Computed tomography revealed a soft tissue density lesion in the middle ear cavity and mastoid antrum with the destruction of the mastoid septae. Magnetic resonance imaging revealed a lesion in the right middle ear cavity associated with pachymeningitis and right sigmoid and transverse sinus thrombosis. Tissue pathology revealed dense plasma cell-rich chronic inflammation with storiform fibrosis. Immunohistochemistry was positive for IgG4. Hence, a diagnosis of IgG4-related disease causing mastoiditis, pachymeningitis and cerebral venous thrombosis was made. The patient was successfully operated and treated with steroids. IgG4-RD remains a rare but serious condition. It is crucial to identify and treat this condition promptly as it can lead to permanent organ damage. When patients continue to experience middle ear symptoms after an infection has been treated and cancer has been ruled out, it is important to consider inflammatory conditions as a differential diagnosis.

## Introduction

Immunoglobulin G4-related systemic disease (IgG4-RSD) is an underrecognized clinical entity characterized by a chronic inflammatory condition that can affect various organs and tissues in the body. It is often associated with an elevated number of IgG4-positive plasma cells, fibrosis and obliterative phlebitis [1]. IgG4-RSD typically affects middle-aged and elderly individuals, with a distinct male predominance. This condition can affect multiple organs, including the pancreas, salivary glands, lacrimal glands, retro-peritoneum, kidneys and lymph nodes, among others. The presentation can vary depending on the organs involved. The hallmark histopathological features include a dense lymphoplasmacytic infiltrate, fibrosis and the presence of IgG4-positive plasma cells. Oftentimes, these plasma cells are scattered diffusely throughout the affected tissue. IgG4-RSD often responds well to corticosteroid therapy. This is sometimes used both for diagnosis (as a positive response to steroids can support the diagnosis) and for treatment. While IgG4-RD is rare, its true prevalence might be somewhat underrepresented due to diagnostic challenges. It is also vital to recognize and treat this disorder before it progresses to a poorly responsive fibrotic disease. With this case report, we aim to create a greater awareness of this disease and how a methodical diagnostic approach can gradually lead to more accurate identification and understanding of this condition.

## Case presentation

**Figure 1:**
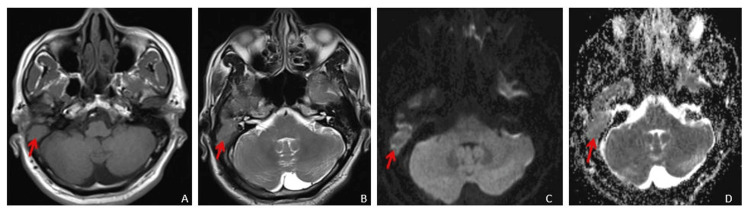
MRI brain axial images (A - T1WI, B - T2WI, C - DWI, D - ADC) showing soft tissue intensity lesion in right middle ear cavity and mastoid air cells causing destruction of the outer mastic cortex, showing minimal diffusion restriction on DWI with low ADC values. MRI: Magnetic resonance imaging; DWI: diffusion-weighted imaging; ADC: apparent diffusion coefficient; T1WI: T1-weighted image; T2WI; T2-weighted image

A 53-year-old male patient came to our institute with a right-sided hemicranial headache for the past 10 days and diplopia on the right side during eye abduction. No previous history of headache or any other illness was present. Magnetic resonance imaging (MRI) brain plain and contrast, 2D time of flight (TOF) MR venography and contrast MR venography were done. A hyperintense lesion was observed in the middle ear cavity on the right side and mastoid air cells on T2-weighted imaging (T2WI) (Figure 1B) and fluid-attenuated inversion recovery (FLAIR), accompanied by erosion and destruction of the outer mastoid cortex showing mild diffusion restriction on diffusion-weighted imaging (DWI) (Figure 1C) with corresponding low apparent diffusion coefficient (ADC) values (Figure 1D). 

**Figure 2:**
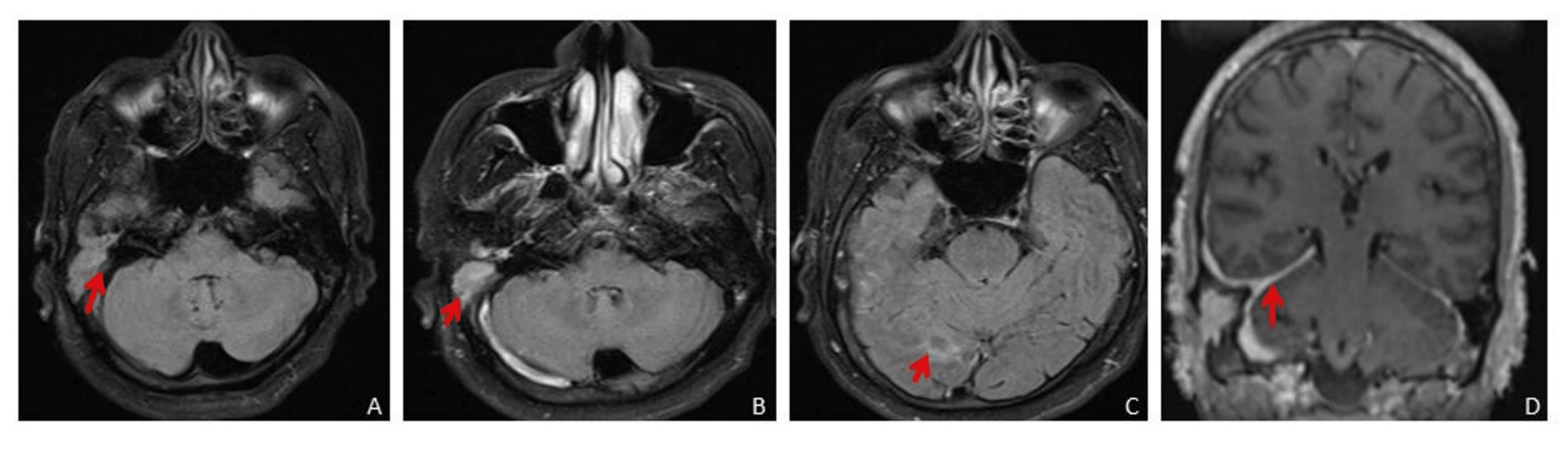
(A) Axial FLAIR (plain) showing slightly hyperintense soft tissue intensity lesion in the right middle ear cavity and mastoid air cells. (B) Axial post-contrast FLAIR showing enhancing lesion in the right middle ear cavity and mastoid air cells. (C) Axial post-contrast FLAIR showing leptomeningeal enhancement in sulcal spaces in the right temporal and occipital region. (D) Coronal post-contrast T1FS showing thickening and dense enhancement of the right leaf of tentorium cerebelli, pachymeningeal enhancement in the right temporal region, enhancing lesion in the right middle ear cavity and mastoid air cells with erosion of the outer mastoid cortex. FLAIR: Fluid-attenuated inversion recovery; T1FS: T1-weighted fat-saturated

A contrast study showed dense patchy meningeal enhancement in the right temporo-occipital region and along the right leaflet of tentorium cerebelli. Mild leptomeningeal enhancement was noted in the right occipital region (Figure 2).

**Figure 3:**
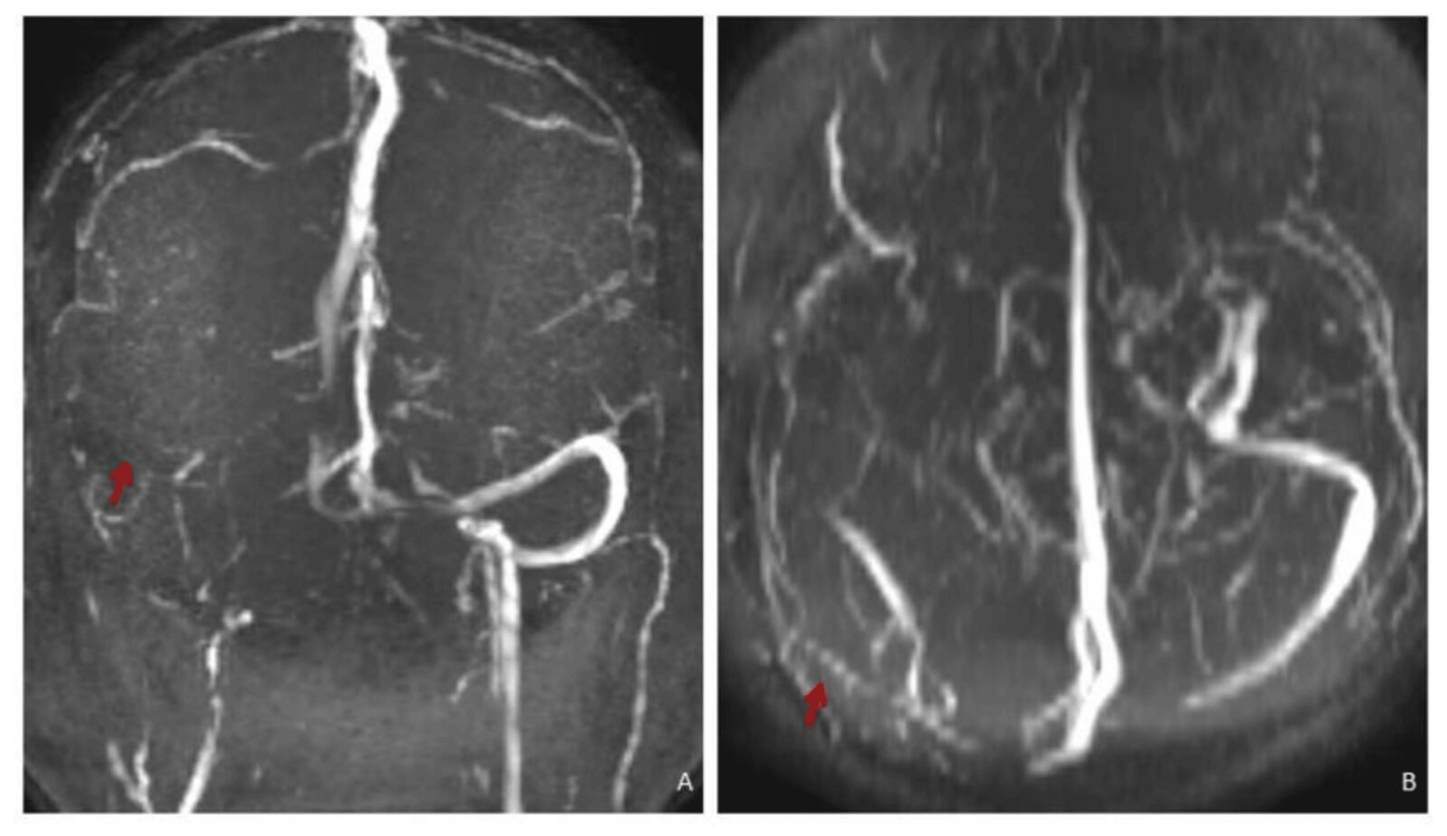
MR 2D TOF MR venography MIP images: (A) Coronal and (B) axial showing loss of flow-related signal in the right transverse and sigmoid sinus suggestive of dural venous sinus thrombosis. TOF: Time of flight; MIP: maximum intensity projection

Few white matter ischaemic foci were noted in the right fronto-parietal white matter appearing hyperintense on T2WI and FLAIR. There was a loss of flow void in the right transverse and sigmoid sinus on T2WI appearing bright on FLAIR - either slow flow or venous sinus thrombosis. Other dural venous sinuses showed normal flow void. No venous haemorrhagic or non-haemorrhagic infarct was noted. 2D time of flight (TOF) MR brain venography showed loss of flow in the right transverse and sigmoid sinus (Figure 3).

**Figure 4:**
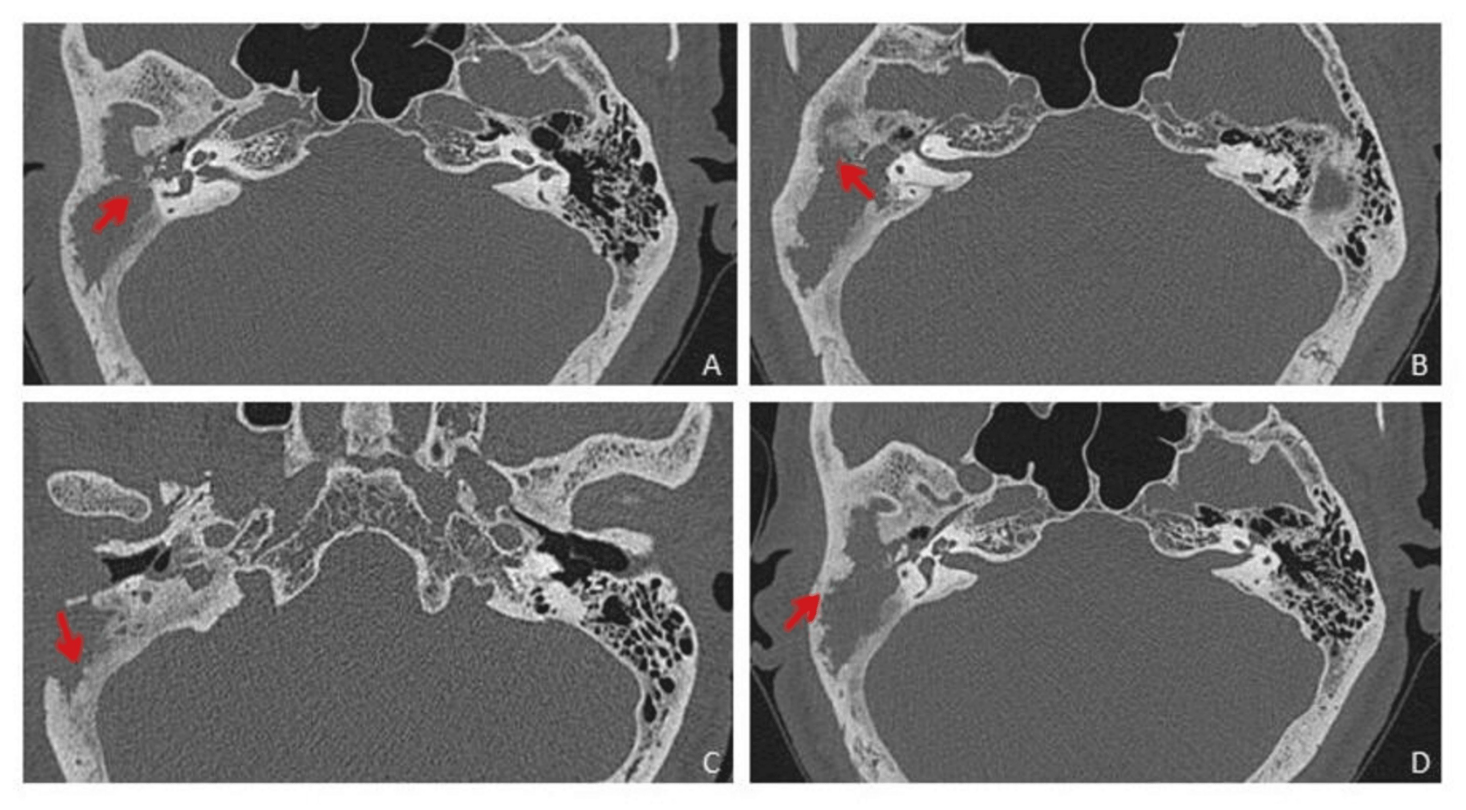
HRCT temporal axial images showing soft tissue density lesion in the right middle ear cavity and mastoid air cells with the destruction of the mastoid septae, outer mastoid cortex and adjoining bony external auditory canal. HRCT: High-resolution computed tomography

Contrast MR brain venography showed filling defect in the right transverse and sigmoid sinus suggestive of dural venous sinus thrombosis. Other dural venous sinuses and deep venous system appeared normal. The possibility of cholesteatoma in the right mastoid with right transverse and sigmoid sinus thrombosis and early meningitis was considered. High-resolution computed tomography (HRCT) of the temporal bone revealed a large soft tissue density lesion in the right middle ear cavity, mastoid antrum and mastoid air cells with the destruction of the mastoid septae (Figure 4).

**Figure 5:**
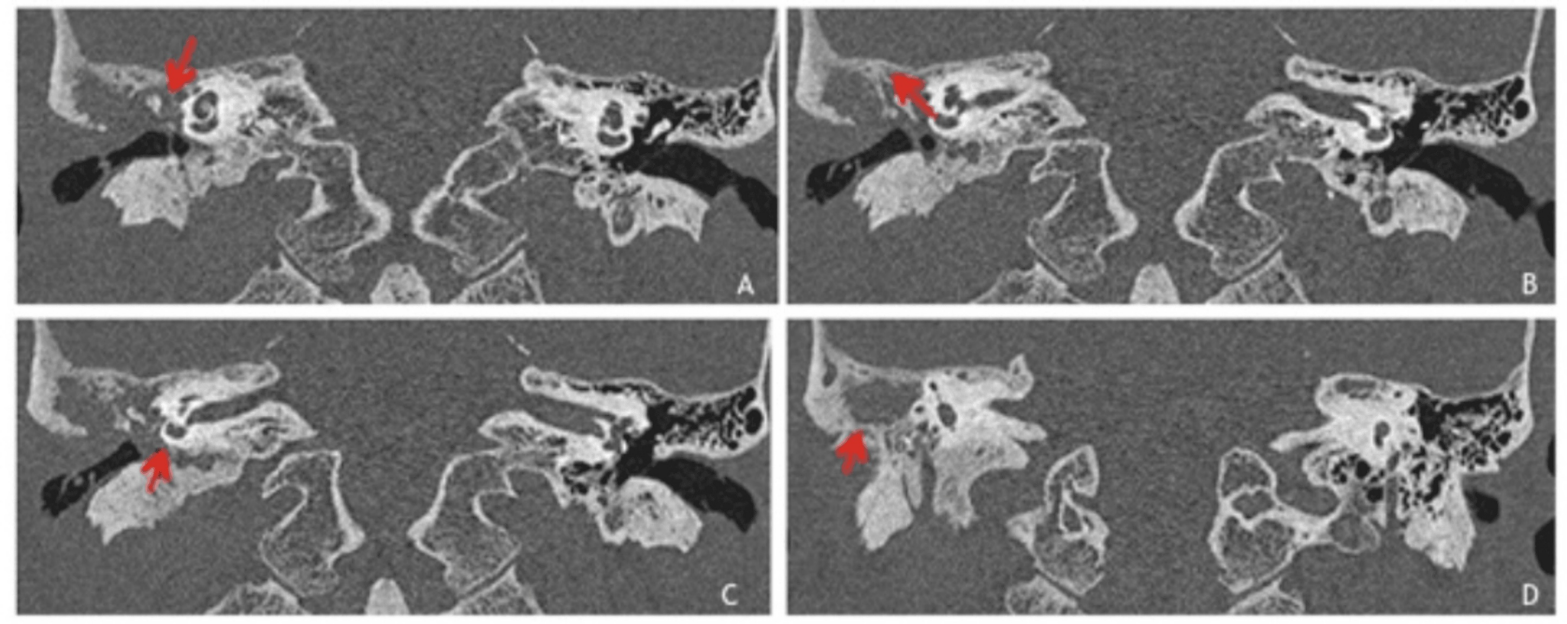
HRCT temporal coronal images showing soft tissue density lesion in middle ear cavity (in epitympanum, mesotympanum and hypotympanum, and Prussack’s space) and mastoid air cells with the destruction of the mastoid septae, outer mastoid cortex and adjoining bony external auditory canal and causing thinning and erosions of tegmen tympani. HRCT: High-resolution computed tomography

The lesion was extending in epitympanum, mesotympanum and hypotympanum, and Prussak's space. Subtle erosions of the incus were noted. The erosion and destruction of the roof of the bony portion of the external auditory canal, scutum and outer mastoid cortex were noted with rarefaction of the rest of the mastoid (Figure 5).

**Figure 6:**
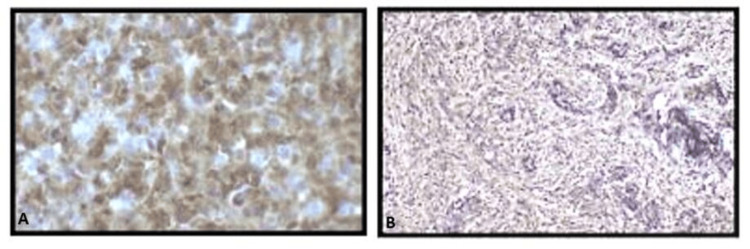
Immunohistochemical staining: (A) IgG4 (MRQ 44) diffusely positive; (B) IgG (367 M-18) diffusely positive. IgG4: Immunoglobuin G4

Inner ear structures appeared normal. Extension of soft tissue was noted in the external auditory canal, and the possibility of aggressive cholesteatoma or neoplastic aetiology was considered. Haemogram, urine and liver function tests (LFTs) were normal. Cerebrospinal fluid (CSF) showed no growth, white blood cells (WBCs) were 0-1 per high power field, red blood cells (RBCs) were 0-2 per high power field. No organism was seen. Ophthalmic examination revealed bilateral papilloedema and right sixth nerve palsy. The patient also complained of right-side tinnitus. In view of the right complicated chronic suppurative otitis media (CSOM) with right transverse and sigmoid sinus thrombosis, right intact canal wall-modified radical mastoidectomy with ossicular reconstruction and type IV tympanoplasty with partial mastoid obliteration was done. The biopsy from the mastoid cavity of the right ear showed dense plasma cell-rich chronic inflammation with storiform fibrosis. IgG immunohistochemistry revealed the presence of immunoglobulin IgG subclass 4 (IgG4) (Figure 6).

Hence a diagnosis of IgG4-related disease causing mastoiditis, pachymeningitis and cerebral venous sinus thrombosis was made. The patient was treated with a high dose of steroids and showed nervous and otologic improvement, following which he was discharged and scheduled for regular follow-ups at our institute.

## Discussion

Immunoglobulin G4-related disease (IgG4-RD) is a relatively newly recognized immune-mediated condition characterized by elevated levels of serum immunoglobulin G4 (IgG4) and tissue infiltrates of IgG4-positive plasma cells [2]. In 2001, Hamano et al. published a seminal paper reporting elevated serum levels of IgG4 in patients with autoimmune pancreatitis [3]. The recognition of raised IgG4 serum levels of individuals with autoimmune pancreatitis played a significant role in establishing IgG4-RD as a systemic disease affecting various organs. Subsequent research has further characterized the clinical features, histopathology and treatment responses associated with IgG4-RD, encompassing not only the pancreas but also other affected organs. In 2004, Dr. Kamisawa proposed the term 'IgG4-related autoimmune disease' to describe the broader spectrum of diseases associated with IgG4 and the involvement of multiple organs beyond the pancreas. This term emphasized the systemic nature of the condition, leading to the recognition that various diseases previously deemed 'idiopathic' or of unknown origin were part of the clinical spectrum of IgG4-related disease (IgG4-RD) [4]. While IgG4-RD primarily affects non-neurological tissues, there have been reported cases of nervous system involvement, although such cases are considered rare. Mikulicz's disease (now often considered a manifestation of IgG4-RD) complicated by autoimmune hypophysitis is an example of neurological involvement [5].

In our case, the histological findings of dense plasma cell-rich chronic inflammation with storiform fibrosis and positive immunostaining of tissue for IgG4 led to the diagnosis of IgG4-RD.

The exact prevalence of IgG4-RD is challenging to determine precisely due to several factors. One significant factor is the relative novelty of the recognition of this condition, and it has only gained broader awareness in recent years. Additionally, IgG4-RD can manifest in various organs and tissues, leading to diverse clinical presentations. The involvement of different systems in the body makes it challenging to diagnose and may result in underreporting or misdiagnosis [6].

The diagnosis of IgG4-RD is a complex process involving a combination of clinical, imaging, serologic, histologic and immunohistochemical features. While various elements contribute to the diagnosis, histopathology has been established as the gold standard. International guidelines, formulated by experts, provide specific criteria for the pathologic diagnosis of IgG4-RD.

The key histologic features that contribute to an absolute diagnosis of IgG4-RD include the following: dense lymphoplasmacytic infiltrate, storiform-type fibrosis and obliterative phlebitis. The presence of these three features together strongly supports the diagnosis of IgG4-RD. It is, however, essential to note that the absolute count of IgG4-positive plasma cells, though important, aren't sufficient alone for the diagnosis on their own. Therefore, elevated IgG4-positive plasma cells must be considered in the context of the overall histologic features [7].

## Conclusions

As we overcome diagnostic challenges with a collaborative radiological, clinical and pathological algorithm, our knowledge of IgG4-related disease (IgG4-RD) continues to expand as new case reports emerge. Its identification is now providing explanations and potential treatments for previously poorly understood diseases. The involvement of the middle ear and mastoid in IgG4-RD is uncommon, and this manifestation poses diagnostic challenges. Chronic infectious conditions and even malignancies affecting the mastoid can sometimes mimic the histologic features seen in IgG4-RD. Extensive fibrosis associated with this condition can lead to irreversible organ damage, hence clinicians must consider this rare condition when diagnosing unusual and persistent ear problems, even if there are no other noticeable symptoms. Given the complexity of the diagnostic process, a collaboration between clinicians, radiologists and pathologists is often necessary to ensure an accurate and timely diagnosis, enabling appropriate management and treatment of IgG4-RD.
